# Inhibition of CD4+CD25+ Regulatory T Cell Function and Conversion into Th1-Like Effectors by a Toll-Like Receptor-Activated Dendritic Cell Vaccine

**DOI:** 10.1371/journal.pone.0074698

**Published:** 2013-11-11

**Authors:** Major K. Lee, Shuwen Xu, Elizabeth H. Fitzpatrick, Anupama Sharma, Holly L. Graves, Brian J. Czerniecki

**Affiliations:** Harrison Department of Surgical Research, Hospital of the University of Pennsylvania, Philadelphia, Pennsylvania, United States of America; Saint Louis University School of Medicine, United States of America

## Abstract

Despite the success of vaccines against some microbial pathogens, their utility in the prevention and treatment of cancer has thus far been limited. We have previously demonstrated that vaccination with dendritic cells activated with the TLR-4 ligand LPS and IFN-γ promotes an antigen-specific anti-tumor response that prevents tumor recurrence. To evaluate this mechanistically, we here studied the effects of this TLR-activated DC on regulatory T cell activity. Dendritic cells activated with LPS and IFN- γ negated the effects of regulatory T cells on responder cell proliferation. Restoration of responder cell proliferation was noted when TLR-activated dendritic cells were separated from both regulators and responders by a semi-permeable membrane. The effect is therefore mediated by a soluble factor but was independent of both IL-6 and IL-12. Furthermore, the soluble mediator appeared to act at least in part on the regulators themselves rather than responder cells exclusively. Because recent studies have demonstrated conversion of T regulatory cells into IL-17-producing effectors, we further questioned whether the TLR-activated dendritic cell would induce cytokine production and effector function in our system. We found that regulators produced a substantial amount of IFN- γ in the presence of TLR-activated dendritic cells but not immature dendritic cells. IFN-γ production was associated with upregulation of the Th1 transcriptional regulator T-bet, and a significant fraction of IFN-γ-producing regulators coexpressed T-bet and FoxP3. While the effects of the LPS-activated dendritic cell on responder cell proliferation were IL-12 independent, upregulation of T-bet was inhibited by a neutralizing anti-IL-12 antibody. Collectively, these and prior data suggest that varying innate immune signals may direct the phenotype of the immune response in part by inhibiting suppressor T cells and promoting differentiation of these regulators into particular subsets of effectors.

## Introduction

Dendritic cells act as surveyors highly active in antigen uptake, processing, and presentation, and they are chiefly responsible for the sensitization of naïve T cells [1]–[3]. Recently, the role of the dendritic cell in the initiation of the immune response has been magnified through the discovery of pattern recognition receptors [4], [5]. It is now clear that presenting cells bear receptors (including Toll-like receptors [TLR]) that recognize generalized molecular patterns shared by various classes of microorganisms. Signaling through Toll-like receptors activates the immune response through multiple mechanisms; Toll ligands not only activate presenting cells, but also inhibit regulatory cells that otherwise suppress the adaptive response. Most notably, signaling through Toll-like receptors TLR-2, TLR-4, TLR-8, and TLR-9 has been shown to reverse suppression by immunoregulatory CD4+CD25+Foxp3+ T cells (referred to here as T_regs_) [6]–[11].

A proposed breakthrough for anti-tumor vaccines was the utilization of tumor antigen-bearing dendritic cells. Given their central role in initiating immunity, administration of dendritic cells bearing tumor peptides carries the potential to generate a vigorous tumor-specific immune response. Dendritic cells have been used as immunotherapeutics in multiple clinical trials with varying success, and ideal strategies for activating, targeting, and delivering these cells are not yet fully elucidated [12].

We have previously detailed our clinical results using a TLR-4-activated dendritic cell vaccine to engender an antigen-specific immune response and prevent recurrence of HER-2/*neu*-positive ductal carcinoma in situ [13]. Given that Toll signals have been shown to inhibit T_reg_ function, we hypothesized that the clinical efficacy of this vaccine may derive in part from its effects on regulatory T cells. Here, we demonstrate that the TLR-activated dendritic cell vaccine not only inhibits T_reg_ effects on responder cells but also converts the regulators themselves into IFN-γ-producing effectors. Both effects occur via soluble mediators, but distinct signals appear to govern T_reg_ inactivation versus conversion into Th1-like effectors. While the capacity of TLR signaling to inhibit T_reg_ function has been shown, conversion into Th1-like cells has not been demonstrated clearly. Elucidating the mechanism of this Toll-activated dendritic cell vaccine raises new perspectives regarding how T_regs_ are integrated into global immunity and illustrates a property potentially desirable in the development of future immunotherapies.

## Materials and Methods

### Ethics Statement

Approval for the use of human tissue was obtained from the University of Pennsylvania Office of Regulatory Affairs Institutional Review Board. Written informed consent was obtained from patients whose tissue was used in the study.

### Preparation of human PBMC fractions

Healthy donor males and females provided informed consent and were leukapheresed. Blood product was then elutriated to obtain monocyte-rich and lymphocyte-enriched fractions that were cryopreserved as described previously [14].

### Preparation of dendritic cells

To generate immature dendritic cells, monocytes were cultured at 3×10^6^/mL in a 1 mL volume of monocyte-macrophage serum-free medium containing 50 ng/mL GM-CSF for approximately 48 hours at 37°C. To generate monocyte-derived, LPS-activated dendritic cells, monocytes were again cultured at 37°C in serum-free medium containing GM-CSF. Approximately 24 hours later, IFN-γ (1000 units/mL) was added. Approximately 24 hours after addition of IFN-γ, LPS was added at 10 ng/mL for 6 hours and cells were then harvested.

### CFSE Labeling

Cells were harvested and resuspended at a density of 1×10^7^ cells per mL in Iscove's Modified Dulbecco's Medium or PBS. An equal volume of 5 mM CFSE in IMDM (or PBS) was added and cells were incubated at 37°C for 5 minutes. The reaction was quenched through the addition of an equal volume of heat inactivated human serum. Labeled cells were washed twice and resuspended in culture medium (IMDM+5% human serum) for *in vitro* stimulations.

### Flow Cytometric Analysis

Cell suspensions were prepared in FACS buffer (PBS+3% FCS+0.01% azide), and anti-human CD4 APC (BD Pharmingen, San Jose, CA) and anti-human CD11c PE (BD Pharmingen) antibodies were used for analysis. Flow cytometric analysis was performed on a Becton Dickinson Immunocytometry System (San Jose, CA) FACSCalibur cytometer. Data processing was accomplished with Becton Dickinson CellQuest Pro™ software.

### Intracellular Staining

For intracellular staining of IFN-γ, cells were harvested following co-culture and restimulated in 50 ng/mL PMA (Sigma-Aldrich) and 250 ng/mL ionomycin (Sigma-Aldrich) along with Golgistop™ (BD Pharmingen) for 4 hours. Cells were then stained with antibodies to surface markers in FACS Buffer for 30–60 minutes. Afterward, cells were washed with PBS, harvested, and permeabilized by incubation in Fixation/Permeabilization working solution (eBioscience, San Diego, CA) for 30–60 minutes as per manufacturer's protocol. Cells were washed in Permeabilization Buffer and then stained with anti-IFN-γ (BD Pharmingen) as per manufacturer's protocols. Cells were then washed and analyzed by flow cytometry. Intracellular staining using anti-FoxP3 (236A/E7 and PCH101, eBioscience) and anti-T-bet (BD Pharmingen) was conducted in similar fashion excepting that there was no restimulation with PMA/ionomycin.

### FACS Purification of Cell Populations

Cells were sorted on a BD FACSVantage SE high-speed cell sorter with FACSDiVA Option (BDBiosciences, San Jose, CA). The three-laser Vantage is equipped with 5W argon, mixed gas argon-krypton, and air-cooled helium-neon lasers. Cells were stained with anti-human CD4 FITC and anti-human CD25 PE (BD Pharmingen). Sorted cells were gated on the CD4 positive, CD25 positive or CD4 positive, CD25 negative populations. Forward scatter pulse width (FSC-W) was used as an additional gated parameter to exclude cell aggregates. Purity checks on the sorted populations exceeded 99%.

### ELISA assay

2.5×10^5^ FACS-sorted CD4+CD25+ T cells were co-cultured with 2×10^5^ immature or LPS-activated DC1 dendritic cells along with 1 mg/mL anti-CD3 (BD Pharmingen) in 0.5 mL total volume at 37°C for 5 days. At the end of 5 days, supernatants were harvested and analyzed for cytokine production by ELISA. Capture and biotinylated detection antibodies and standards for IFN-γ and IL-17 (BD Pharmingen) were used according to the manufacturer's recommendations and protocols.

### Statistics

P values between groups were calculated using a student T test. A P value less than 0.05 was considered statistically significant.

## Results

### CD4+CD25+ T cells inhibit responder cell proliferation in the presence of immature but not DC1 dendritic cells

We have previously demonstrated that tumor antigen-bearing dendritic cells generated using IFN-γ and the TLR-4 agonist LPS (referred to here as DC1 dendritic cells) promote a targeted immune response in patients with ductal carcinoma in situ [13]. Prior studies have consistently demonstrated that TLR agonists including LPS are capable of inhibiting suppression mediated by CD4+CD25+Foxp3+ regulatory T cells [6]–[11]. Thus, we hypothesized that the response to this TLR-activated dendritic cell vaccine occurs at least in part through downregulation of T_reg_-mediated immunosuppression.

**Figure 1:**
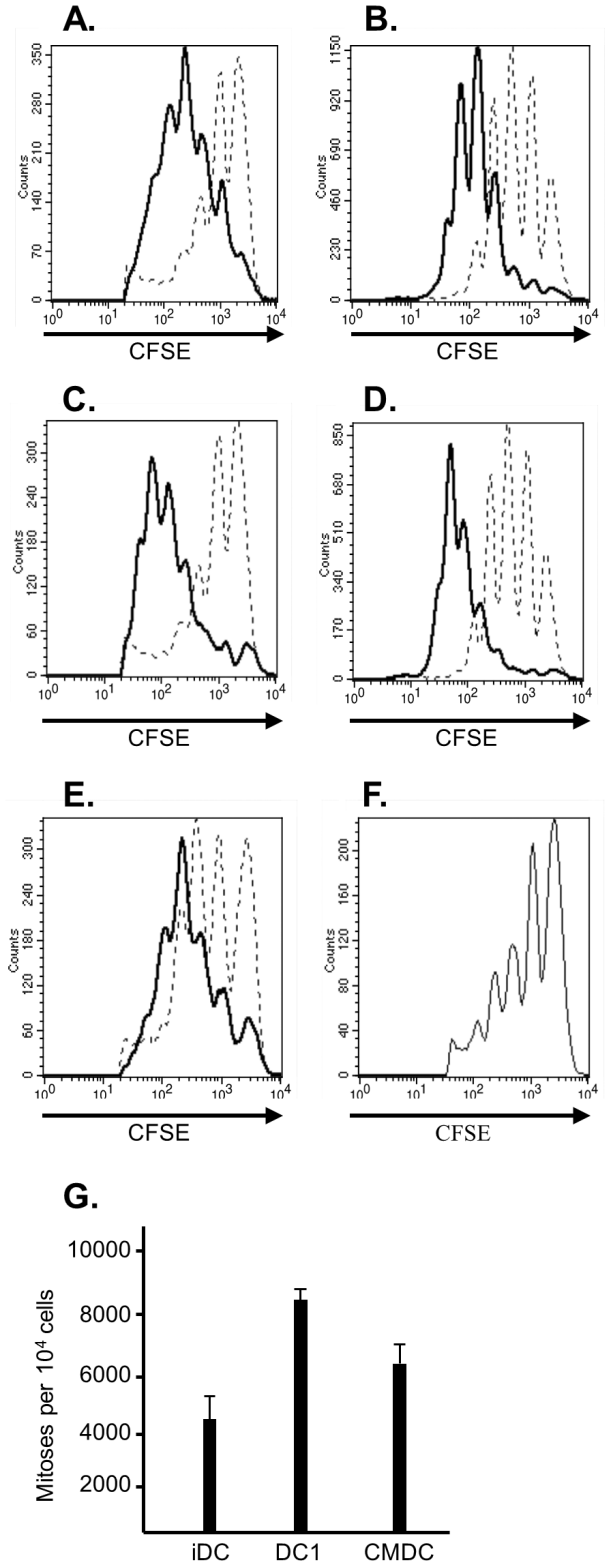
T_regs_ inhibit responder cell proliferation in the presence of immature but not DC1 dendritic cells. (*1A&B*) 2.5×10^5^ CFSE-labeled unfractionated responder lymphocytes were initially co-cultured with 1×10^5^ immature dendritic cells in the presence (dashed line) or absence (solid line) of 1.25×10^5^ sorted, purified CD4+CD25+ T cells for 5 days. Responder cell proliferation is shown for CD4-gated (*1A*) or CD8-gated (*1B*) T cells. Data shown are representative of at least 10 experiments. (*1C&1D*) 2.5×10^5^ CFSE-labeled unfractionated responders were co-cultured with 1.25×10^5^ sorted CD4+CD25+ T cells and 1×10^5^ immature (dashed line) or DC1 dendritic cells (solid line). Again responder cell proliferation is shown for CD4-gated (*1C*) or CD8-gated (*1D*) T cells. Data shown are representative of at least 10 experiments. (*1E*) 2.5×10^5^ CFSE-labeled unfractionated responders were co-cultured with 1×10^5^ dendritic cells matured using a conventional cytokine maturation cocktail (IL-1, IL-6, TNF-α, PGE-2) in the presence (dashed line) or absence (solid line) of 1.25×10^5^ sorted CD4+CD25+ T cells. Proliferation for CD4-gated T cells is shown (*N* = 4). (*1F*) Proliferation of CD4-positive responders in the presence of T_regs_ and immature dendritic cells treated briefly with LPS (15 minutes) prior to co-culture is shown (*N* = 3). (*1G*) A mathematical algorithm previously applied elsewhere was used to calculate the number of mitoses per 10^4^ responder CD4-gated T cells in the presence of iDC, DC1, or conventional cytokine maturation cocktail DC (CMDC).

To test this hypothesis, we compared the capacity of human CD4+CD25+ T cells to inhibit the proliferation of CD4 and CD8 lymphocytes to TCR stimulation (anti-CD3) in the presence of immature dendritic cells (iDC) versus DC1 dendritic cells. 1.25×10^5^ sorted CD4+CD25+ T cells were combined with 2.5×10^5^ CFSE-labeled unfractionated lymphocytes (CD4 and CD8 positive) and 1×10^5^ immature dendritic cells. We found that the proliferative response of both CD4 and CD8 positive T cells at day 5 was inhibited in the presence versus the absence of T_regs_ (Figure 1A&B). Proliferation of sorted CD4+CD25− T cells was similarly inhibited excluding the possibility that this result was purely an artifact of using unfractionated responders (data not shown). By contrast, inclusion of DC1 dendritic cells matured using IFN-γ and LPS restored the proliferation of both CD4 and CD8 positive T cells despite the presence of regulators (Figure 1C&D). Responder proliferation in the presence of T_regs_ and DC1 dendritic cells was similar to that in the absence of the regulatory population. To quantify these differences, we capitalized on the successive halving of fluorescence intensity characteristic of CFSE to calculate the number of mitoses per 10^4^ cells as has been done elsewhere [15], [16]. The DC1 population significantly increased proliferation in the presence of regulators (Figure 1G; *P*<.001 for DC1 vs iDC). Notably, dendritic cells matured using a conventional cytokine-based maturation cocktail (IL-1, IL-6, TNF-α, PGE_2_) did not fully restore proliferation of effectors in the presence T_regs_ (Figure 1E,1G). Responder cell proliferation in the presence of DC1 versus these cells approached but did not quite reach statistical significance (*P* = 0.087 for DC1 versus cytokine-matured DC).

That LPS used to mature the DC1 dendritic cells contaminated the co-culture causing the observed effect was excluded by brief pretreatment of iDC with LPS (15 minutes). Suppression was unaffected in this setting, indicating that the DC1 population needed to be formally matured and contaminating LPS was not responsible for the result (Figure 1F). These results collectively demonstrate that DC1 dendritic cells inhibit T_reg_-mediated suppression of both CD4 and CD8-positive T cells responding to anti-CD3.

### Inhibition of T_reg_ function by DC1 dendritic cells is not due to apoptosis

We next attempted to characterize the mechanism by which the DC1 vaccine inhibits T_reg_-mediated suppression. First we assessed whether the vaccine inhibits T_reg_-mediated suppression by inducing apoptosis in the regulatory T cell complement. The abundance of evidence collectively suggests that T_reg_ sensitivity to apoptosis is defined in part by the surrounding microenvironment including both TCR signals and cytokines present [17]. These environmental factors are admittedly simplified in this *in vitro* system. However, the ability to distinguish T_regs_ from other cell populations in this assay allows us to more clearly evaluate whether T_reg_ apoptosis is significantly increased in the presence of the DC1 vaccine.

**Figure 2:**
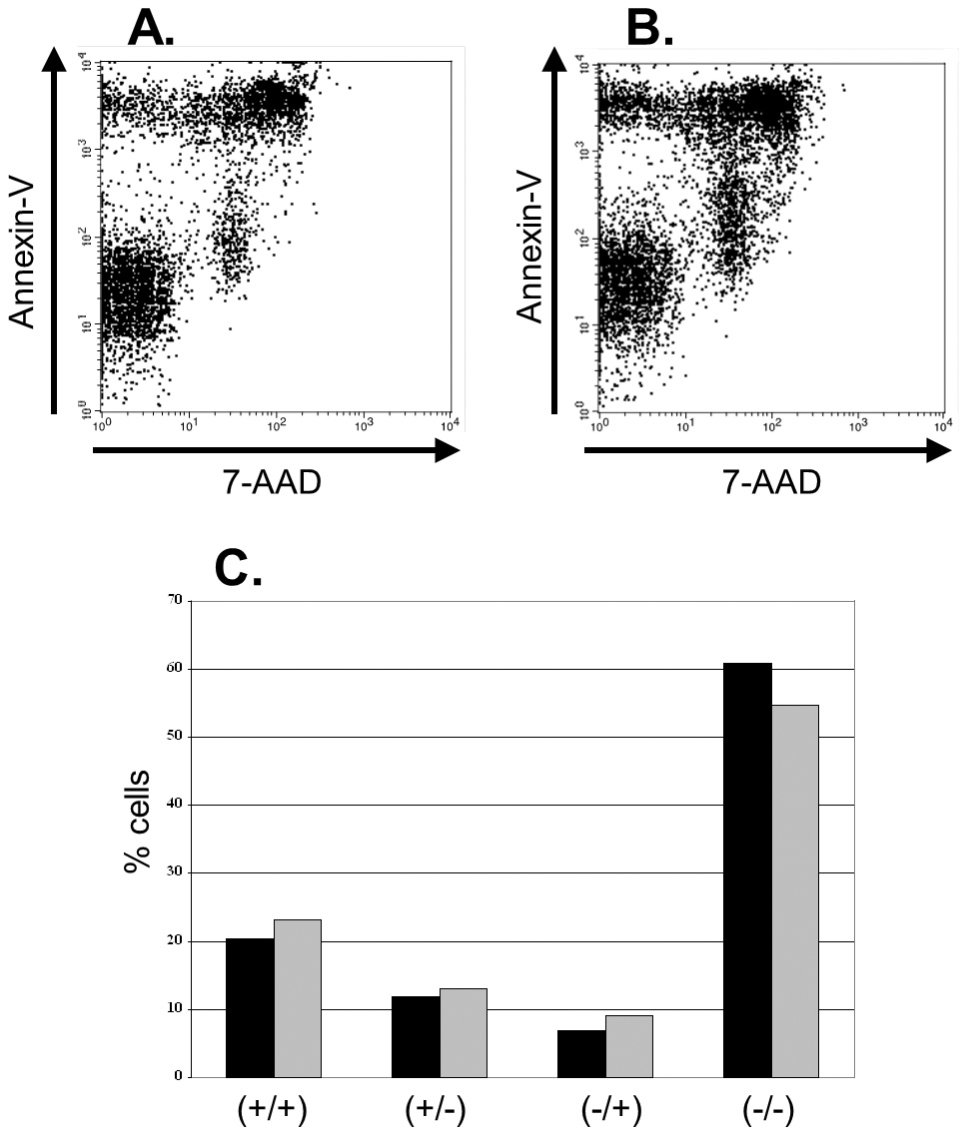
Inhibition of T_reg_ function by DC1 dendritic cells is not due to apoptosis. 1.25×10^5^ sorted, purified CD4+CD25+ T cells were co-cultured with 1×10^5^ immature dendritic cells (*2A*) or DC1 dendritic cells (*2B*). Expression of the apoptotic markers Annexin-V and 7-AAD 24 hours later is shown. Figure 2C summarizes the percent of cells expressing both markers (+/+), Annexin-V only (+/−), 7-AAD only (−/+), or neither marker (−/−). (iDC black, DC1 gray; *N = 4*).

To test whether DC1 dendritic cells induce cell death to neutralize T_regs_, we co-cultured sorted CD4+CD25+ T cells with immature versus DC1 dendritic cells and compared the expression of the apoptotic markers Annexin-V and 7-AAD after 24 hours. We chose 24 hours as our time point with the presumption that proliferative differences noted at day 5 would result from apoptotic events occurring far earlier. After 24 hours of culture, we found that the expression of both Annexin-V and 7-AAD was similar amongst T_regs_ co-cultured with either dendritic cell population (Figures 2A and 2B; *P*>0.2 for the Annexin^+^/7-AAD^+^ and Annexin^−^/7AAD^−^ groups). These data suggest that DC1 dendritic cells do not significantly alter T_reg_ apoptosis as compared with immature dendritic cells. Thus, the break in suppression in their presence likely results from other effects.

### Inhibition of T_reg_ function by DC1 dendritic cells results from a soluble factor but is IL-6 and IL-12 independent

**Figure 3:**
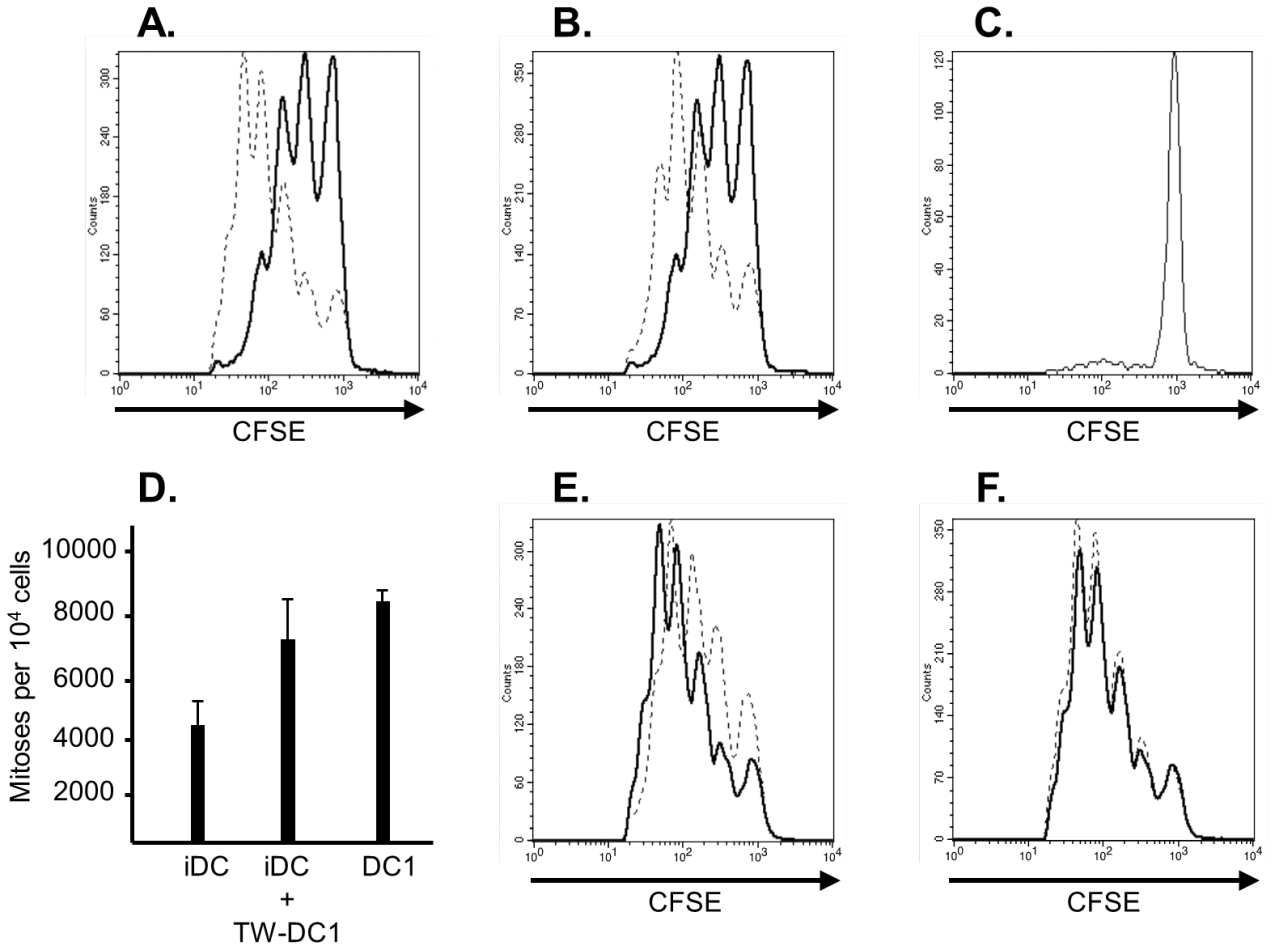
Inhibition of T_reg_ function by DC1 dendritic cells results from a soluble factor. (*3A*) 1.25×10^5^ sorted, purified CD4+CD25+ T cells were co-cultured with 2.5×10^5^ CFSE-labeled unfractionated responder lymphocytes and 1×10^5^ immature dendritic cells (solid line) or DC1 dendritic cells for 5 days (dashed line). Data shown are gated on CD4-positive cells and are representative of at least 10 experiments. (*3B*) 1.25×10^5^ T_regs_ were co-cultured with 2.5×10^5^ CFSE-labeled responders and 1×10^5^ immature dendritic cells for 5 days (solid line). In addition, 1×10^5^ DC1 dendritic cells were added to a transwell membrane placed in the culture well (dashed line). Data shown are gated on CD4-positive cells and are representative of 4 experiments. (*3C*) 1.25×10^5^ T_regs_ were co-cultured with 2.5×10^5^ CFSE-labeled responders alone for 5 days. 1×10^5^ DC1 dendritic cells were added to a transwell membrane placed in the culture well. Data shown are gated on CD4-positive cells (*N* = 2). (3*D*) The number of mitoses per 10^4^ cells is summarized for CD4-positive responder cells in the presence of T_regs_ and iDC alone, iDC with DC1 added to the Transwell membrane, and DC1 alone. (*3E&F*) 1.25×10^5^ T_regs_ were co-cultured with 2.5×10^5^ CFSE-labeled responders and 1×10^5^ DC1 dendritic cells in the presence (dashed line) or absence (solid line) of neutralizing anti-IL-12 (*3E*) or anti-IL-6 (*3F*) antibodies (5 µg/mL). Data shown are gated on CD4-positive cells and are representative of at least three experiments.

Having established that the effect of DC1 cells on T_reg_-mediated suppression is likely not due to apoptosis, we next questioned whether this effect was cell-contact dependent or mediated by soluble factors. To do so, we again co-cultured CFSE-labeled effector cells with unlabeled CD4+CD25+ T cells in the presence of immature dendritic cells. This time we added alternate dendritic cell populations separated by a semi-permeable Transwell® membrane. When an additional complement of immature dendritic cells was added to the Transwell® membrane, there was no effect on suppression in the presence of iDC. However, when the DC1 population was added to the Transwell® membrane, we observed a break in T_reg_ mediated suppression that was similar to that seen when T_regs_ were co-cultured directly with DC1 (Figure 3B). To control for the possibility that DC1 dendritic cells themselves migrated through the Transwell® to break suppression, we co-cultured regulatory T cells and CFSE-labeled effectors in the absence of dendritic cells but added DC1 cells separated by the membrane. Very little proliferation was noted (Figure 3C). Quantitatively, the proliferative response noted when DC1 cells were added to the Transwell membrane differed significantly from that seen in the presence of iDC alone and approached that observed when DC1 were added directly to the coculture. In fact, there was no significant difference in proliferation when DC1 were added to the Transwell® versus directly to the co-culture. (Figure 3D; *P* = 0.037 for iDC alone versus iDC with DC1 in Transwell®; *P* = 0.15 for DC1 alone versus iDC with DC1 in Transwell®). These data collectively suggest that the DC1 population can inhibit T_reg_-mediated suppression in cell contact-independent fashion.

In our view, the two most likely soluble mediators for the break in suppression are IL-12 and IL-6. The DC1 population secretes a large amount of IL-12 which is principally involved in Th1 immunity [18], while IL-6 has previously been shown to be central to LPS-mediated T_reg_ inhibition *in vitro* [9]. We therefore tested whether neutralization of IL-6 or IL-12 would restore the inhibitory effects of T_regs_ in the presence of DC1 dendritic cells. We found that the proliferation of effectors in the presence of T_regs_ and DC1 dendritic cells was minimally affected by inclusion of neutralizing antibodies to IL-12 and IL-6 (Figures 3E, 3F). Taken together with the Transwell® experiments, these data suggest that a soluble factor other than IL-6 and IL-12 mediates the break in suppression effected by DC1 cells.

### DC1 dendritic cells inhibit T_regs_ directly

The preceding experiments are unable to discern whether the DC1 population acts on the regulators themselves or simply releases responder cells from T_reg_ inhibition. That the DC1 effect is mediated by a soluble factor allows us to test whether the vaccine affects T_regs_ directly or licenses responders to act despite their presence. Specifically, we questioned whether pre-treatment of regulators or responders with media taken from DC1 dendritic cells would reverse T_reg_-mediated suppression.

**Figure 4:**
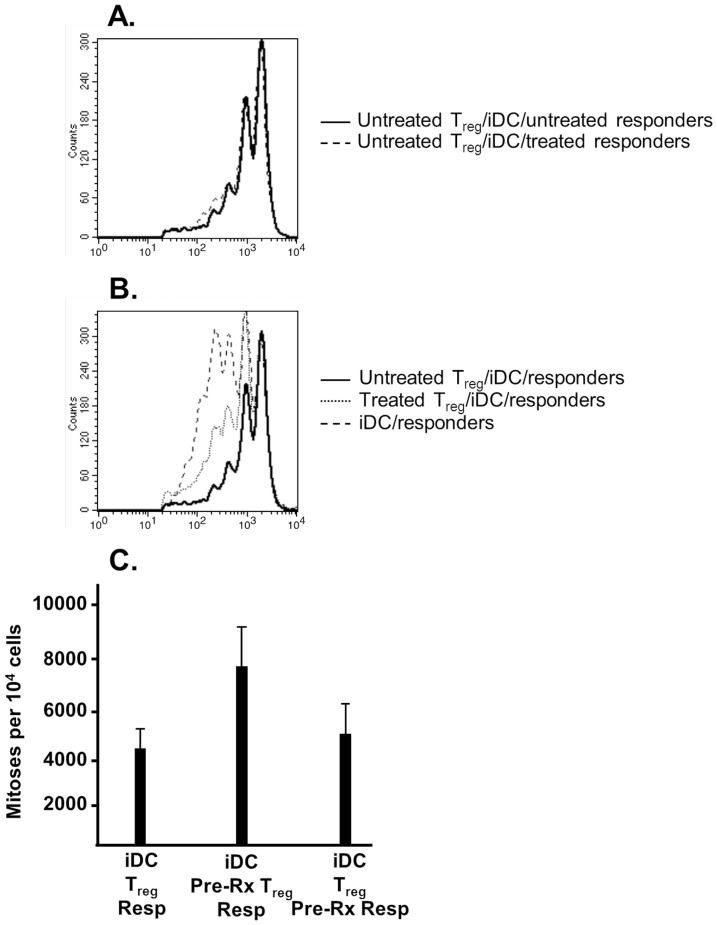
DC1 dendritic cells inhibit T_regs_ directly. DC1 dendritic cells were generated as previously described. Medium from these cells was then harvested and combined 1∶1 with culture medium to create the “pretreatment” medium. CD4+CD25+ T cells or effectors were cultured in the pretreatment medium for 24 hours at a concentration of 3×10^6^ cells/mL. These “treated” cells were then harvested, washed, and used in cocultures as previously described. (*4A*) 2.5×10^5^ “treated” or “untreated” CFSE-labeled unfractionated responder lymphocytes were co-cultured with 1×10^5^ immature dendritic cells and 1.25×10^5^ sorted, purified CD4+CD25+ T cells for 5 days. CD4-positive responder cell proliferation is shown. (*4B*) 2.5×10^5^ CFSE-labeled unfractionated responder lymphocytes were co-cultured with 1×10^5^ immature dendritic cells and 1.25×10^5^ sorted, purified “treated” or “untreated” CD4+CD25+ T cells for 5 days. CD4-positive responder cell proliferation is shown. (*4C*) The number of mitoses per 10^4^ cells is summarized. In each case, data shown are representative of three separate experiments.

We first cultured sorted CD4+CD25+ T cells or CFSE-labeled effector cells in equal parts culture medium and medium transferred from DC1 dendritic cell cultures. Presumably, the soluble mediator that inhibits T_reg_ function is present in medium taken from DC1 cultures and thus will act on T_regs_ or responders during this “pre-treatment.” These cells were harvested and washed 24 hours later and then utilized in co-cultures. We found that pre-treating responders with DC1 media had no effect on T_reg_-mediated suppression (Figure 4A). However, pre-treatment of the regulators prior to co-culture somewhat restored responder cell proliferation in their presence (Figure 4B). Our quantitative analysis confirmed the significance of the effect (Figure 4C; *P* = 0.04 for proliferation in the presence of untreated T_regs_ versus “pre-treated” T_regs_; *P* = 0.256 for proliferation in the presence T_regs_ and untreated versus “pre-treated” responders). This finding suggests that a soluble factor is released by DC1 dendritic cells and acts at least in part on the regulators themselves to break suppression.

### Suppressor CD4+CD25+ T cells secrete effector cytokines in the presence of DC1 dendritic cells

Recent studies in several experimental models have shown that dendritic cells of various phenotypes are capable of converting regulatory T cells into antigen-specific autoimmune effectors [19], [20]. Mechanistically, this typically involves downregulation of the transcriptional regulator FoxP3 and upregulation of effector cytokines. Most consistently noted is conversion of T_regs_ into IL-17-producing effector cells that likely mediate Th17 immunity [19]–[21]. We thus questioned whether the break in suppression noted here reflects simple deactivation of regulatory T cells or their conversion into effectors. Given that DC1 dendritic cells exhibit robust production of IL-12 and are strong inducers of Th1 immunity, we hypothesized that they would more likely secrete IFN-γ than IL-17.

**Figure 5:**
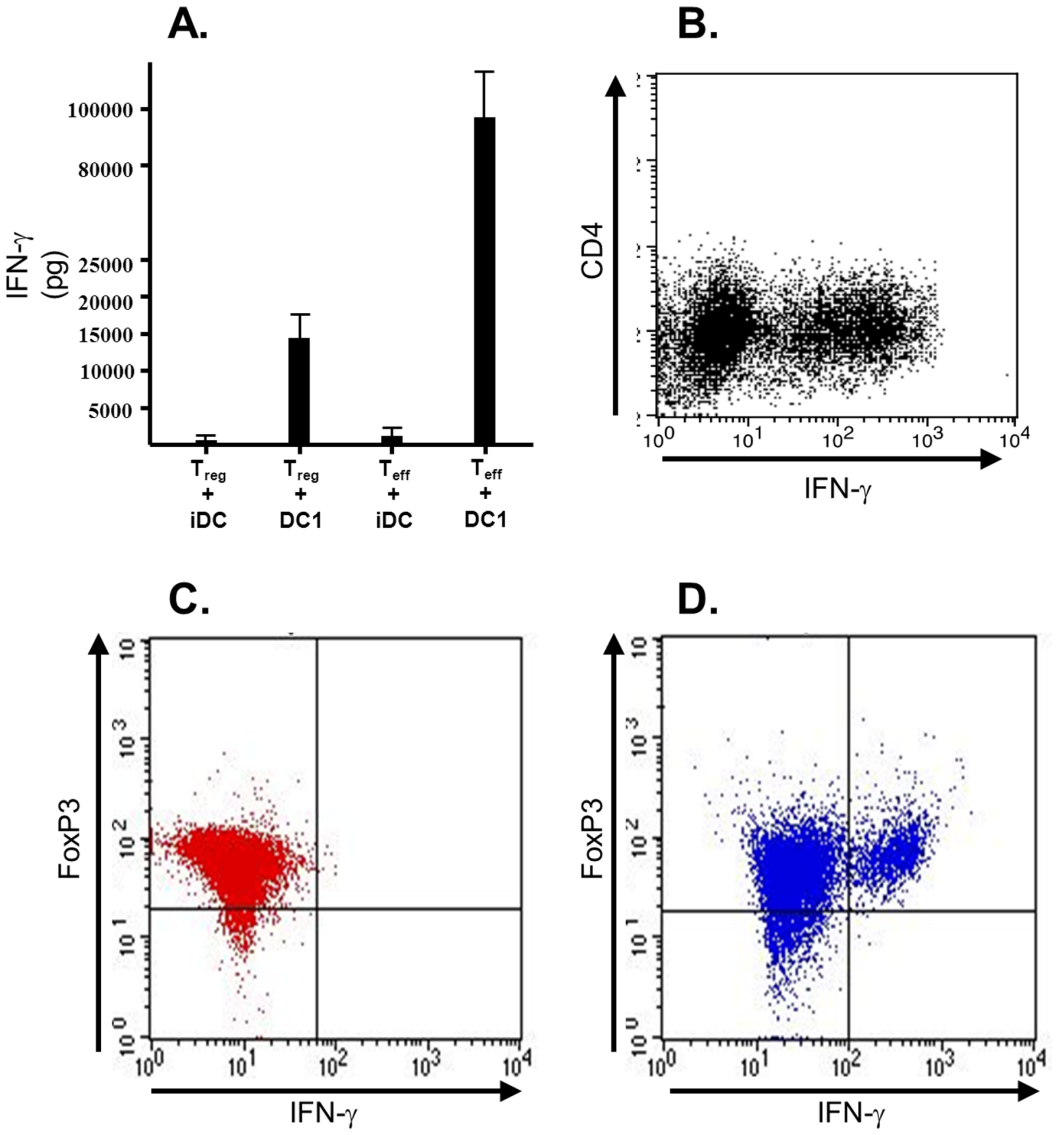
Suppressor CD4+CD25+ T cells secrete effector cytokines in the presence of DC1 dendritic cells. (*5A*) 2.5×10^5^ sorted CD4+CD25+ (T_reg_) or CD4+CD25− (T_eff_) T cells were combined with 2.0×10^5^ immature or DC1 dendritic cells. Supernatants were harvested at 5 days and ELISA was used to measure the amount of IFN-γ present in the supernatant. Data shown are the average of at least four experiments. (*5B*) At day 5, some culture samples were permeabilized and intracellular IFN-γ was detected by flow cytometry. (*5C&D*) 2.5×10^5^ sorted CD4+CD25+ T cells were cocultured with immature (*5C*) or DC1 (*5D*) dendritic cells; intracellular expression of FoxP3 and IFN-γ was detected in permeabilized cells 5 days later.

To test our hypothesis, we co-cultured T_regs_ or CD4+CD25− effectors with immature versus DC1 dendritic cells at the typical 1.25∶1 ratio and measured cytokine production using ELISA. We found that both T_regs_ and effectors co-cultured with immature dendritic cells made essentially no IFN-γ. However, both populations made detectable amounts of IFN-γ when co-cultured with DC1 dendritic cells (Figure 5A). To validate that the cytokine measured was produced by the T cells and not the dendritic cell complement, we harvested cells following co-culture and evaluated the intracellular production of IFN-γ. We found that a significant fraction of CD4+ T cells were IFN-γ-positive (Figure 5B). By contrast, a much smaller number of CD11c-positive dendritic cells were cytokine-positive (data not shown). The purity of the sorted CD4+CD25+ population is reliably >99%, ruling out the possibility that cytokine production is mediated by contaminating cells.

A fair percentage of the sorted CD4+CD25+ population is FoxP3-negative (approximately 20%). It is therefore plausible that the FoxP3+ cells to which suppression is best ascribed are simply deactivated here and that cytokine production instead comes predominantly from this FoxP3-negative cohort. To exclude this possibility, we cocultured CD4+CD25+ T_regs_ with immature versus DC1 dendritic cells and simultaneously evaluated intracellular expression of both FoxP3 and IFN-γ. We found that a considerable fraction of regulators cocultured with DC1 dendritic cells coexpressed both FoxP3 and IFN-γ. That FoxP3 negative cells were exclusively responsible for cytokine production is therefore unlikely (Figure 5D).

### CD4+CD25+ T cells upregulate T-bet in the presence of DC1 dendritic cells

Recent studies indicate that conversion of T_regs_ to effector cells is correlated with downregulation of the transcriptional regulator FoxP3 and upregulation of a variety of cytokines [19], [20]. The DC1 population is known to polarize a Th1 immune response due to its robust production of IL-12 which fosters development of IFN-γ-producing T cells [18]. Differentiation of Th1 cells is programmed through the action of several transcription factors including the T box factor T-bet. We therefore hypothesized that conversion of T_regs_ to IFN-γ-producing effector cells in this study may be associated with downregulation of the T_reg_-specific transcription factor FoxP3 and upregulation of T-bet.

**Figure 6:**
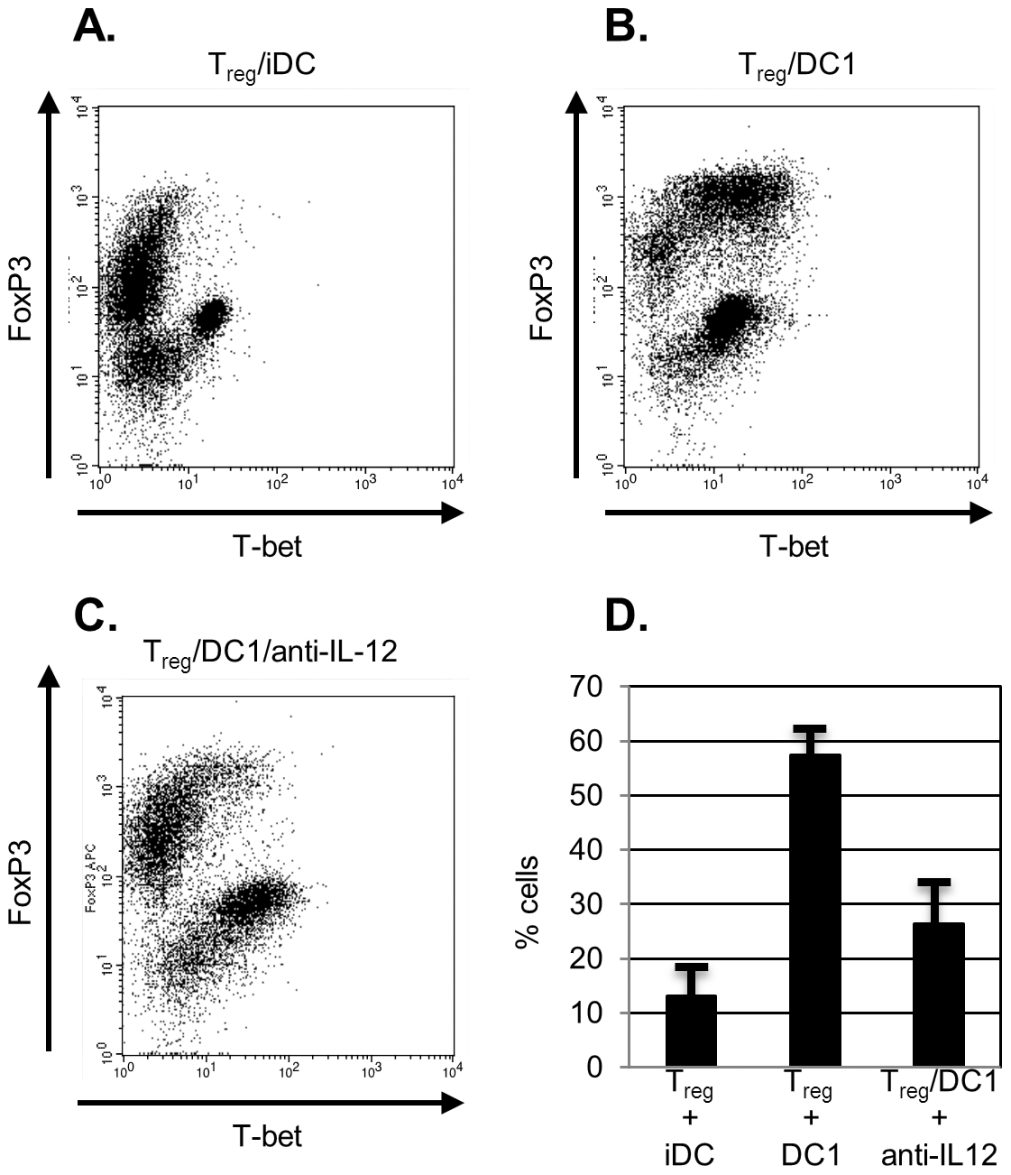
CD4+CD25+ T cells upregulate T-bet in the presence of DC1 dendritic cells. 1.25×10^5^ CD4+CD25+ T cells were co-cultured with 1×10^5^ immature (*6A*) or DC1 (*6B,C*) dendritic cells. Neutralizing anti-IL-12 antibody (5 µg/mL) was included in some samples (*6C*). At 48 hours cells were harvested, permeabilized, and intracellular expression of T-bet and FoxP3 was detected by intracellular staining. Data shown are gated on CD4-positive cells and are representative of at least three separate experiments in each instance. Figure 6D summarizes the percent of FoxP3+ cells that are T-bet positive at 48 hours for each group.

To test this premise, we co-cultured CD4+CD25+ regulators with immature or DC1 dendritic cells and soluble CD3 then used intracellular cytokine staining to analyze expression of FoxP3 and T-bet. As compared with immature dendritic cells, DC1 dendritic cells induced upregulation of T-bet amongst T_regs_ (Figure 6A, 6B). A significantly greater fraction of FoxP3-positive cells were noted at day 2 to express T-bet in the presence of the LPS-activated DC (Figure 6D; *P* = 0.01 for DC1 versus iDC). CD4+CD25+ T cells incubated in the presence of immature dendritic cells did not upregulate T-bet, and very few cells were measured as double positive. We again chose a slightly earlier time point (day 2) under the presumption that differences in proliferation and cytokine production noted at day 5 reflect earlier changes in transcription factor expression.

These data show that production of IFN-γ by CD4+CD25+ T cells co-cultured with DC1 is correlated with upregulation of T-bet especially amongst FoxP3-positive cells. These data also further discount the possibility that cytokines detected by ELISA are generated by expanding FoxP3 negative CD4+CD25+ T cells. In our view, the fact that the majority of T-bet-positive cells are also FoxP3-positive suggests that regulatory T cells are converted into cytokine-producing effectors; less likely but not completely excluded is that FoxP3 negative cells both upregulate FoxP3 and become unilaterally responsible for the cytokine detected. That T_regs_ transitioning to effectors at least transiently express multiple transcriptional regulators has been reported in conversion to Th17 cells [21], [22].

Because IL-12 is critical to Th1 differentiation and subsequent production of IFN-γ, we questioned whether the conversion of regulators to IFN-γ-producing cells noted here was IL-12 dependent. Although we have shown that DC1 dendritic cells negate the effects of T_regs_ on responder cell proliferation in IL-12-independent fashion, it is feasible that different signals govern conversion to effectors. We therefore co-cultured T_regs_ with DC1 dendritic cells in the presence of a neutralizing anti-IL-12 antibody and evaluated expression of FoxP3 and T-bet. In contrast to our prior observation, we found that the neutralizing antibody did inhibit T-bet upregulation which was reduced in its presence (Figure 6C). There was no significant difference in T-bet upregulation when the neutralizing antibody was included (Figure 6D; *P* = 0.21 for iDC versus DC1/anti-IL-12)

## Discussion

We have previously detailed our clinical results employing a monocyte-derived, LPS-activated, HER-2/*neu*-expressing dendritic cell immunotherapeutic agent against HER-2/*neu*-positive ductal carcinoma in situ [13]. Prior studies have demonstrated that signals through a variety of Toll-like receptors including TLR-4 are capable of inhibiting regulatory T cells. Thus, we here hypothesized that our dendritic cell vaccine engenders a potent immune response in part by deactivating regulatory T cells.

We first demonstrated that CD4+CD25+ T cells inhibit effector T cell proliferation in the presence of immature dendritic cells. We then illustrated a break in T_reg_-mediated suppression in the presence of the DC1 vaccine, as CFSE-positive effectors exhibit robust proliferation despite the presence of the regulatory complement. This effect occurred across a semi-permeable membrane and thus was cell contact-independent. The use of CFSE and more specifically the labeling of effector cells alone is a central aspect of this experimental model. Proliferation assays used to demonstrate suppression in many other studies suffer from their reliance on tritiated thymidine. Numerous reports demonstrate that regulatory T cells are not completely anergic and in fact have significant proliferative potential especially in the context of inflammatory signals [23], [24]. Assays based on tritiated thymidine cannot account for regulatory cell proliferation making the data difficult to interpret. Our CFSE-based assay tracks proliferation specifically amongst effector cells and directly compares their proliferation in the presence/absence of T_regs_ and varying dendritic cell populations. In doing so, it eliminates the possibility that proliferation by CD4+CD25+ regulators promotes misinterpretation of results.

A number of recent studies have demonstrated that a variety of Toll-like receptors including TLR-2, TLR-4, TLR-8, and TLR-9 can abrogate T_reg_-mediated suppression [6]-[11]. Perhaps of most relevance to our own study is that of Pasare and Medzhitov [9]. These authors demonstrated that dendritic cells activated with LPS secrete soluble factor(s) that release effector cells from suppression by CD4+CD25+ T cells. IL-6 was required for this effect but appeared to act synergistically with one or more other cytokines. Our results are largely compatible with these findings but extend them in a number of respects. First, our study was conducted using human cell populations including a dendritic cell currently in use in immunotherapy. This translates the prior findings directly to the clinical venue. Secondly, while these authors added LPS directly to dendritic cells to induce their effect, our study shows that dendritic cells activated with LPS then transferred to co-cultures containing T_regs_ and effectors remain capable of breaking suppression. This finding indicates an enduring effect on the APC and suggests prolonged secretion of a soluble mediator(s) capable of inhibiting regulators. Further, the suggestion that LPS breaks T_reg_-mediated suppression by acting on the suppressor population or effectors directly is excluded here. Rather, it is the LPS-activated dendritic cell that mediates the effects. Third, our study demonstrates an effect which is IL-6 and IL-12-independent but appears mediated by a soluble factor. It is plausible that this factor synergizes with IL-6 in the aforementioned study. Lastly, our data indicate that the T_reg_ population here is not only temporarily deactivated, but may also contribute to developing immunity through conversion into T-bet-positive, IFN-γ-secreting effectors.

The latter finding in concert with those of other groups suggests a new paradigm for the integration of regulatory T cells into the immune milieu. T_regs_ are currently viewed as principal mediators of peripheral tolerance that control inflammatory immune responses and prevent autoimmunity. How a productive immune response is initiated despite the presence and activity of these cells is not entirely certain. Studies demonstrating T_reg_ inhibition in the context of inflammation suggests one model—that inflammatory signals encountered in the setting of pathogenic insult deactivate regulators to initiate immunity. Of note in this regard is our finding that a TLR-activated dendritic cell restores proliferation of responder cells despite the presence of T_regs_. Recent studies illustrating conversion of T_regs_ into antigen-specific Th17 cells extend this model by suggesting that T_regs_ may play an active role in the immune response rather than simply bystanding [19]–[22]. Our data support this postulate as well. However, in our model, the Toll-activated dendritic cell predominantly secretes IFN-γ and thus better reflects a Th1 cell. Interestingly, this finding unlike the effect noted on the proliferative response was IL-12-dependent. Collectively, these data raise several possibilities regarding the function of regulatory T cells in the developing immune response. First is that distinct signals direct cessation of T_reg_ activity versus conversion into cytokine-producing effectors. It is plausible that signals present during the initiation of the immune response determine its magnitude in part by deciding whether T_regs_ bystand passively or contribute as cytokine-producing effectors. Second is that T_regs_ may be converted into various subsets of antigen-specific effectors (Th1, Th2, Th17) depending on the type of immune response mandated. On a molecular basis, this may occur through the upregulation of lineage-specific transcription factors (e.g. T-bet), and cells may at least transiently express high levels of factors that direct both the regulatory and the effector phenotype. These cells would then act synergistically with similarly differentiated, liberated FoxP3-negative cells to maximize immunity. That IFN-γ-secreting T_regs_ may still remain in a regulatory role dampening Th1 responses through direct contact and/or inhibitory cytokines has also been observed [25], [26]; this could still occur while proliferative responses are nonetheless licensed. Regardless, how T_reg_ function is restored to temper the immune response upon pathogen eradication remains a mystery. Previous studies have shown that T_regs_ activated to produce IL-17 may retain suppressive capacity if removed from the inflammatory environment [21].

Our results also carry import regarding the design of tumor immunotherapy vaccines. Regulatory T cells have proven problematic in attempts to induce tumor immunity since the 1980s [27]. Given the potential significance of T_regs_ in immunotherapy, several recent protocols combine anti-tumor vaccines with agents that deplete T_regs_. These combined approaches often exhibit greater benefit than either agent alone [28]–[34]. Thus, maximizing vaccine efficacy may require subversion of regulatory processes. Notably, several recent studies have shown that a variety of vaccination strategies increase the frequency and/or potency of regulatory T cells [35]–[37]. Perhaps most notably, dendritic cells matured using a conventional cytokine cocktail were found to expand FoxP3^high^ cells that appear to have suppressor function [38]. This may compromise the development and endurance of tumor immunity. By contrast, the DC1 dendritic cell vaccine is ideal in that it appears to inhibit rather than activate T cell-mediated suppression and may carry the added benefit of converting these cells into tumor-reactive effectors. In our model, T_reg_ inhibition in the presence of the DC1 vaccine was not observed in the presence of dendritic cells matured using a conventional protocol. Although there has been no direct comparison of the DC1 vaccine to dendritic cells matured using conventional protocols, the effects on T_reg_ function demonstrated here may render it more effective and may account in part for the findings noted in the clinical venue.
